# Assessing right ventricular function in pulmonary hypertension patients and the correlation with the New York Heart Association (NYHA) classification

**DOI:** 10.18632/oncotarget.19026

**Published:** 2017-07-05

**Authors:** Xiaoke Shang, Shuna Xiao, Nianguo Dong, Rong Lu, Lijun Wang, Bin Wang, Yousan Chen, Liang Zhong, Mei Liu

**Affiliations:** ^1^ Department of Cardiovascular Surgery, Union Hospital, Tongji Medical College, Huazhong University of Science and Technology, Hubei Province 430222, China; ^2^ Department of Pediatric Intensive Care Unit, Hubei Maternal and Child Health Hospital, Hubei Province 430070, China; ^3^ Department of Intensive Care Unit, Wuhan No.1 Hospital, Tongji Medical College, Huazhong University of Science and Technology, Hubei Province 430222, China; ^4^ Department of Intervention, Wuhan Asia Heart Hospital, Hubei Province 430022, China; ^5^ Department of Radiology, Wuhan General Hospital of CPLA, Guangzhou Military Command, Hubei Province 430070, China; ^6^ National Heart Centre Singapore, 169609, Singapore; ^7^ Duke NUS Medical School, 169857, Singapore

**Keywords:** right ventricular, pulmonary hypertension (PH), New York Heart Association (NYHA), PV Loop

## Abstract

This investigation aimed to compare the pressure-volume loop (PV loop) measurements in three less symptomatic categories (New York Heart Association classes , NYHA I, II, and III) of pulmonary hypertension (PH) patients since NYHA classification system performance is limited by the shortcomings discussed above.

Thirty-six patients were enrolled in this study with PV loop measurement acquisition via micro-conductance catheters. Functional classification according to NYHA was determined with comprehensive assessing function and activity. Catheterization and MRI was applied to obtain variables on right ventricle (RV) functions. Correlation test was applied to test the relationship between measured PV loop measurements and NYHA classification.

A group of PV loop measurements, including end-systolic pressure (RVESP) RV end-diastolic pressure (RVEDP), and RV arterial elastance (RVEa), are well correlated with three NYHA classes (I, II, and III). Moreover, RVESP and RVEa significantly correlated with two groups of NYHA classes (I and II/III) while RVEDP, RV end-diastolic volume (RVEDV), and RV end-systolic volume (RVESV) significantly moderately correlated with two groups of NYHA classes (I/II and III). This study suggests the promising role of PV loop analysis in assessing functional capacity in progressive but less symptomatic PH patients.

## INTRODUCTION

Pulmonary Hypertension (PH), defined by a mean pulmonary artery pressure ≥ 25 mm Hg, is a progressive disorder and a fatal disease with only a 58% survival rate at 3 years [1]. It is a type of high blood pressure that affects the pulmonary vascular system, primarily the small pulmonary arterioles. Though the initial syndrome of PH involves the pulmonary vasculature, this disease normally leads to hypertrophy of the right ventricle and reduced cardiac output, eventually causing right heart failure or death [2, 3]. Chronic heart failure remains a serious and burdensome healthcare issue, and carries a poor prognosis. The New York Heart Association (NYHA) classification system was first developed in 1928 and most recently revised in 1994 [4]. Despite this powerful prognostic ability, NYHA classification remains an approximate and subjective system by definition; it seems to perform well in more symptomatic patients in classes III and IV, but becomes more subjective when there are fewer symptoms and can make it challenging to consistently classify patients between classes II and III, or between classes I and II [5, 6]. However, this classification should not be abandoned as it provides a rapid assessment of patients’ functional status in everyday clinical practice and is a well established means of predicting prognosis when applied to dichotomously divided patients [5–8]. It is therefore suggested that functional capacity assessment is, fundamentally, an overwhelmingly important prognostic element [5] that could overcome NYHA's limitations in less symptomatic PH patients.

The gold standard for detailed ventricular functional capacity is the pressure–volume (PV) relationship that measures the total mechanical energy of the ventricle and its efficiency as a heat engine and pump, which allows myocardial contractile efficiency to be determined [9]. First proposed by Frank [10] and later thoroughly studied by Suga et al in the 1970s [9, 11, 12], this pressure–volume relationship is capable of determining a considerable amount of information regarding cardiac performance [13]. Conceptually, the pivotal studies using end-systolic and end-diastolic pressure–volume relationships show the ventricular contractility through end-systolic elastance (Ees), the ventricular afterload through arterial elastance (Ea), and the flow output at minimal energy cost (Ees/Ea) [13, 14]. This PV loop analysis is accomplished through non-invasive magnetic resonance imaging (MRI) and invasive catheterization combined in a single heartbeat [13, 15–18]. Since it was first conducted decades ago, this analysis has been widely applied in left ventricular (LV) analysis [19, 20]. However, because right heart failure was thought to be less important and the right ventricle (RV) operates at lower pressures than the left ventricle and the systemic arteries [14], the quantitative analysis and functional prognosis of the right ventricle in PH patients have been less intensively studied than the widely studied left ventricle. The lack of adequate PV loop analysis on RV function in PH patients is at odds with the increasing demands on both clinical management of PH patients and clinical research [21] and hinders the establishment of a combined prognosis based on PH patients’ functional assessment and the popular but subjective NYHA system.

Therefore, this investigation aimed to compare the PV loop measurements in three less symptomatic categories of PH patients (NYHA classes I, II, and III)—categories for which the NYHA classification system performance is limited by the shortcomings discussed above. We also evaluated the prognostic ability of the PV loop measurements in assessing the cardiac functional capacity of PH patients.

## MATERIALS AND METHODS

### Ethics statement and informed consent

With regards to the retrospective nature of this research, the protocol was approved by the review board at Wuhan Asia Heart Hospital. And the authors pledge to abide by the declaration of Helsinki (1996 EDITION). Informed consent was obtained from the parent or legal guardian (age < 18 years) or from the patient (age ≥ 18). Additionally, no intervention was given in participations and the patients’ personal information is being kept confidential.

### Patients and functional classification

Thirty-six patients (with a mean age of 32.81 ± 12.20 years) were enrolled in this study with PV loop measurement acquisition via micro-conductance catheters. The inclusion criteria are based on the RV micro-conductance and Cardiac Magnetic Resonance (CMR) imaging results. The exclusion criteria are: 1) Prothrombin Time International Normalized Ratio (PT-INR test) > 1.6; 2) platelet count < 50,000; 3) patients who are incapable of being performed a surgery with supine position; 4) patients who are allegic to intravenous contrast medium; 5) creatinine (Cr) value greater than 2.0; 6) patients who have atherosclerosis and coronary artery diseases, cancers, diabetes, high cholesterol symptoms, elevated homocysteine levels, metabolic syndrome, and/or blood diseases. The PH diagnosis was established according to the practice guidelines of the American College of Cardiology [22], with the medical history inspection. Functional classification according to NYHA was determined with comprehensive assessing function and activity [4]. It was tried to ensure that the classification would give a comprehensive summary of the patient's clinical condition. The PV loop parameters were not the determinants or the criteria on this classification.

### Right heart catheterization

Catheterization was done in the catheterization laboratory with the patients under conscious sedation and local anesthesia. The following invasive hemodynamic variables were recorded: mean right atrial pressure (mRAP), RV pressure, mean pulmonary arterial pressure (mPAP), and total peripheral resistance (TPR). End-diastolic pressure (EDP) was recorded at the maximal diastolic filling pressure point before the onset of isovolumetric contraction. Diastolic filling pressures were acquired at the minimum pressure point after tricuspid valve opening. End-systolic pressure (ESP) was obtained at the end of the systolic process.

### Cardiac MRI

MRI observation time was approximately 20–30 min, and it took another 20 min for the image processing and recording. RV volumes were calculated with Mass software (MEDIS; Medical Imaging Systems, Leiden, Netherlands) from multiple short axial slice MRI analysis [23]. End-systolic volume (ESV) was referred to as begin-diastolic volume (BDV). End-systolic volume was considered to correspond to BDP and is further referred to as begin-diastolic volume, and end-diastolic volume (EDV) corresponded to EDP. Stroke volume (SV) was calculated from MRI-derived pulmonary artery flow and used to accurately determine RV BDV. RV volumetric filling curves were obtained from the stack of short axis cine images [24, 25].

### Pressure-volume analysis

**Figure 1:**
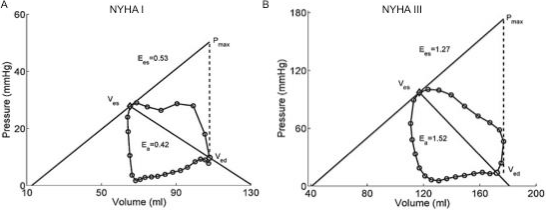
Representative PV loop graphs of NYHA class I patient (Panel A) and NYHA class III patient (Panel B) The PV loop measurements are: (**A**) RVEDV (mL), 107.9; RVESV (mL), 65.3; RVEF (%), 40; Ees, 0.53; Ea, 0.42; Ees/Ea, 1.25; (**B**) RVEDV (mL), 172.1; RVESV (mL), 117.1; RVEF (%), 40; Ees, 1.27; Ea, 1.52; Ees/Ea, 0.84.

The single beat method [26] was employed to calculate elastance-related measurements, i.e., end-systolic elastance (Ees), arterial elastance (Ea), and their ratio (Ees/Ea), as described in [27]; ESP was approximated by mPAP. Ees was then calculated as the slope of end-systolic PV relationship (the difference between maximum pressure (Pmax) and mPAP, all divided by SV), and Ea was estimated by the ratio of mPAP to SV. The single beat method allows for the definition of ESP that is a line drawn from Pmax tangent to the RV pressure–volume loop with relative variation in volume calculated from the integration of pulmonary flow (Figure 1).

### Statistical analysis

Data are expressed as mean ± standard deviation (SD). Statistical significance was defined as two-sided *p* value < 0.05. All statistical analysis was performed with commercially available software (SPSS for Windows, version 19.0; SPSS Inc., Chicago, IL). The Spearman correlation test was applied to test the relationship between measured PV loop measurements and NYHA classification. The NYHA class was predicted by Receiver Operating Characteristic (ROC) analysis. A two-tailed *P* value smaller than 0.05 was considered and applied to all statistical testing as statistically significant.

## RESULTS

### Basic characteristics of the subjects

A total of 36 PH patients (10 male) were enrolled, with a mean age of 32.81 ± 12.20 years and a 6-minute walk distance of 446.42 ± 11.29 meters. Five of the patients were in NYHA class I, 23 were in class II, and 8 were in class III. No severe symptomatic patients (class IV) were included in this study. The patients’ mean pulmonary arterial pressure (mPAP) was 55.19 ± 20.41 mm Hg (range: 10.00 to 92.00). 16.7% of the patients had chest pain, 47.2% had chest distress, 16.7% had hemoptysis, 44.4% had cyanosis, 41.7% had dyspnea, and 33.3% had peripheral edema on their medical history. Overall patient characteristics are summarized in Table 1.

**Table 1:** Patient characteristics (N = 36)

Variable	Mean	SD	Minimum	Maximum
**Clinical data**				
Age (years)	32.81	12.20	7.00	61.00
Height (cm)	157.69	9.95	125.00	174.00
Weight (kg)	50.83	9.95	28.00	74.00
BSA (cm^2^)	1.51	0.18	0.97	1.84
BMI (kg/m^2^)	20.34	2.99	15.05	27.85
BMR (kJ/m^2^/h)	36.94	2.69	32.60	45.30
6-MWD (m)	446.42	11.29	305.00	520.00
HR (bpm)	82.44	14.04	63.00	120.00
**Medical history (%)**				
Chest pain	16.7%			
Chest distress	47.2%			
Hemoptysis	16.7%			
Cyanosis	44.4%			
Dyspnea	41.7%			
Peripheral edema	33.3%			
**Hemodynamic parameters**				
mRAP (mmHg)	7.86	4.79	4.00	25.00
mPAP (mmHg)	73.58	10.42	49.40	92.00
TPR (dyn·s·cm^−5^)	981.06	625.57	78.39	2575.67

Data given as the Mean or Percentage. BSA, body surface area; BMI, body mass index; BMR, basal metabolic rate; 6-MWD, six-minute walk distance; HR (bmp), heart rate (beats per minute); mRAP, mean of right atrial pressure; mPAP, mean of pulmonary arterial pressure; TPR, total pulmonary pressure.

Detailed patient characteristics in different NYHA classes were analyzed and are compared in Table 2. The mean values of the total pulmonary pressure (TRP) and the mean pulmonary arterial pressure (mPAP) in NYHA class II are significantly different from those in class I. In addition, the mean value of right arterial pressure (mRAP) varied monotonically with the classification. These findings demonstrate the NYHA's advantage in PH prognosis. At the same time, though, the 6-minute walk distance, a classic test for heart failure patients, correlated poorly with the classification in PH.

**Table 2:** Comparison of basic characteristics in different NYHA classes

	NYHA I	NYHA II	NYHA III
Group#	1 (*n* = 5)	2 (*n* = 23)	3 (*n* = 8)
Age (year)	32.60 ± 15.73	30.39 ± 10.10	39.88 ± 14.37
Male (%)	40 ± 55	17 ± 39	50 ± 53
Height (cm)	155.20 ± 14.79	156.17± 8.02	163.63 ± 10.73
Weight (kg)	52.80 ± 13.26	49.57 ± 8.82	53.25 ± 11.65
BSA (cm^2^)	1.51 ± 0.24	1.48 ± 0.16	1.57 ± 0.18
BMI (kg/m^2^)	21.66 ± 2.85	20.24 ± 2.88	19.79 ± 3.50
BMR (kJ/m^2^/h)	37.90 ± 4.31	36.78 ± 2.39	36.78 ± 2.65
HR (bmp)	84.80 ± 10.73	81.83 ± 14.02	82.75 ± 17.21
Cardiothoracic ratio	0.51 ± 0.06	0.57 ± 0.09	0.65 ± 0.07*
6-MWD (m)	434.00 ± 88.17	448.14 ± 55.16	452.50 ± 86.57
mRAP (mmHg)	6.25 ± 1.89	6.522 ± 2.61	12.50 ± 7.52
mPAP (mmHg)	30.40 ± 17.24	59.44 ± 17.38**	58.50 ± 21.30*
TPR (dyn·s·cm^−5^)	447.50 ± 242.70	1069.217 ± 549.57*	994.375 ± 895.02

Data given as the Mean ± SD. * ^&^**p < 0.05 ^&^*p <* 0.01, compared to group #1.

### PV loop measurements in different NYHA classes

**Figure 2:**
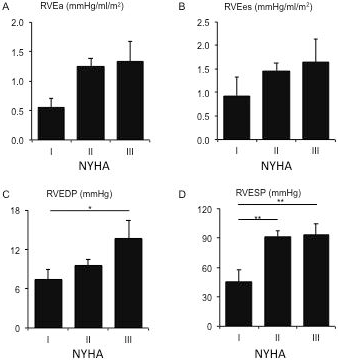
The mean values of RV arterial elastance (RVEa) (Panel **A**), RV end-systolic elastance (RVEes) (Panel **B**), RV end-diastolic pressure (RVEDP) (Panel **C**), and RV end-systolic pressure (RVESP) (Panel **D**) in different NYHA classes.

The PV loop measurements in three NYHA patient groups are summarized in Table 3. As detected in the high NYHA classes (II and III), most of the mean values of the PV loop measurements were accelerated along with the deteriorated PH condition. Meanwhile, the RVEF and RVEes/Ea were inversely related to NYHA classes. Statistical analysis revealed significant differences of RVEDP and RVES*P* values between NYHA class I and those in higher NYHA classes (Figure 2).

**Table 3:** Comparison of PV loop measurements in different NYHA classes

	NYHA I	NYHA II	NYHA III
Group#	1 (*n* = 5)	2 (*n* = 23)	3 (*n* = 8)
RVEDV (ml/m^2^)	144.04 ± 96.66	172.89 ± 90.89	285.10 ± 199.03
RVESV (ml/m^2^)	74.16 ± 50.20	97.35 ± 73.78	184.39 ± 142.42
RVSV (ml/m^2^)	69.90 ± 47.69	75.54 ± 33.63	100.71 ± 75.63
RVEF (%)	48.50 ± 7.35	48.02 ± 15.53	38.55 ± 14.08
LVEDV (ml/m^2^)	92.88 ± 21.54	102.80 ± 46.70	119.05 ± 59.73
LVESV (ml/m^2^)	41.24 ± 20.34	44.79 ± 26.53	61.31 ± 35.82
LVSV (ml/m^2^)	51.66 ± 7.26	58.00 ± 25.58	57.85 ± 28.17
LVEF (%)	57.06 ± 10.37	57.55 ± 10.36	50.53 ± 10.50
RVEDP (mmHg)	7.40 ± 3.36	9.61 ± 4.31	13.75 ± 7.44*
RVESP (mmHg)	45.38 ± 28.01	91.37 ± 29.62**	93.45 ± 30.56**
RVEa (mmHg/ml/m^2^)	0.56 ± 0.34	1.25 ± 0.63	1.33 ± 1.00
RVEes (mmHg/ml/m^2^)	0.93 ± 0.87	1.46 ± 0.80	1.65 ± 1.36
RVEes/Ea	1.58 ± 0.64	1.20 ± 0.48	1.21 ± 0.53

Data given as the Mean ± SD. * ^&^**p < 0.05 ^&^*p <* 0.01, compared to group #1. RVEDV, right ventricular end-diastolic volume; RVESV, right ventricular end-systolic volume; RVSV, right ventricular stroke volume; RVEF, right ventricular ejection fraction; LVEDV, left ventricular end-diastolic volume; LVESV, left ventricular end-systolic volume; LVSV, left ventricular stroke volume; LVEF, left ventricular ejection fraction; RVEDP, right ventricular end-diastolic pressure; RVESP, right ventricular end-systolic pressure; RVEa, right ventricular arterial elastance; RVEes, right ventricular end-systolic elastance.

### PV loop measurements correlated with NYHA classifications

As shown in Table 4, the Pearson correlation test indicated that RVEDV, RVESV, RVEDP, and RVESP significantly correlated (*p* < 0.05) with the three NYHA classifications (I, II, and III). In addition, when the patients were grouped into two NYHA classifications (I and II/III), the classification had a significant association with RVESP and RVEa. When the patients were grouped into NYHA classes I/II and class III, this classification had a significant association with RVEDV, RVESV, and RVEDP. All these results suggest that PV loop measurements, especially RV variables, have significantly moderate correlations with NYHA classification (Figure 2). Right ventricular stroke volume, RVSV (*p* = 0.104) and left ventricular ejection fraction, LVEF (*p* = 0.100) correlated essentially significantly with this grouping.

### ROC analysis of PV loop measurements in NYHA classification

**Figure 3:**
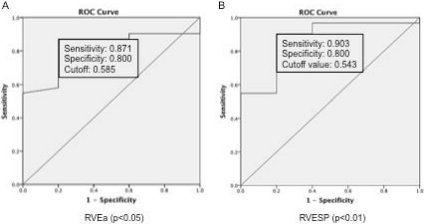
Receiver operating characteristic (ROC) curves of RVEa (Panel **A**) and RVESP (Panel **B**). ROC curves were constructed as plots of sensitivity versus 1 specificity when NYHA classes II and III were considered as positive. The values of sensitivity and specificity were indicated.

Next, we sought to evaluate the diagnostic and prognostic performance of the PV loop measurements in assessing the cardiac functional capacity of PH patients [28]. ROC analysis was applied. Out of the 13 PV loop measurements we tested in correlation analysis, two measurements (RVEa and RVESP) were the most significant discriminators (*p <* 0.05, *p* < 0.01) between both entities with areas under the curve of 0.871 and 0.903 respectively (Figure 3).

## DISCUSSION

Although RV functional evaluation is mainly based on echocardiography technique in clinical practice, magnetic resonance imaging (MRI) appears to be the most accurate method for evaluating RV volume and RVEF [28]. Meanwhile, conductance catheterization represents the gold-standard method for evaluating RV pulmonary coupling measurements [29, 30]. In this study, these two powerful methods were adopted to obtain most hemodynamic and PV loop measurements to assess RV function in PH patients classified by the NYHA system. NYHA classification provides useful and complementary information about the presence and severity of heart failure (HF); it focuses more on exercise capacity and the symptomatic status of the disease [31]. However, this powerful and easily applied classification system has been criticized as an approximate and subjective tool, particularly in the RV functional discrimination between the asymptomatic class I and mildly symptomatic class II [6]. In our study, two PV loop measurements, RVESP and RVEa, correlated significantly (both *p* values less than 0.05) with the grouping of class I, vs classes II and III (Table 4 and Figure 2). In chronic HF brought on by PH, the right ventricle can adapt and remodel in response to the gradual increase in pulmonary vascular resistance (PVR) [32], like the left ventricle facing a progressive increase in systemic vascular resistance [33]. In the asymptomatic and mild phases of PH (in NYHA classes I and II), a homeometric adaptation of RV (i.e., without a chamber dilatation) occurs for the initial increase of RV afterload (RVEa) whose variation is bought by the change of PVR [30]. This adaptation mechanism primarily relies on the homeometric systolic function adaptation. Thus it interprets the good performances of RVEa, RVESP, and RV contractility independent of RV afterload, RVEes/RVEa (RVEes normalized to RV afterload RVEa) on the discrimination of NYHA classification (class I and classes II/III). In this condition, RV and pulmonary are coupling well and RV variables either do not increase (RVEF) or increase slightly (RVSV, RVEDV, RVESV), compensated by the change in RV coupling whose surrogates are RVEes/RVEa and RVEDP [30] (Table 3). Based on this finding, we infer that the transition from class I to class II is probable at the middle or late stage of the RV homeometric adaptation process.

To distinguish patients between class II and III is crucial in a serious, reliable, reproducible assessment for a symptomatic patient because class III is the entry class for the end stage of the PH patient, and special therapeutic interventions are required for this end-stage patient population (i.e., in NYHA classes III and IV) [34–36]. Nevertheless, the NYHA classification's performance in making this distinction has been criticized for its unsatisfying interobserver agreement and its lack of consistency in classification; it is little better than chance [5]. Our finding demonstrates that RVEDV, RVESV, and RVEDP correlated significantly (*p* < 0.05) with the grouping of NYHA classes I/II and class III (Table 4 and Figure 2). RVEF was associated in an indicative significance (*p* = 0.104, data not shown). When homeometric adaptation failure occurs, the systolic function adaptation is unable to afford the deterioration of HF in PH, and RV–pulmonary coupling uncouples, indicated by the maintained RVEes, RVEa, RVEes/RVEa, and RVESP in contrast with increased RV volumes between class II and class III (Table 3). It results in heterometric adaptation, mainly at the cost of the larger increases of RVEDV and RV wall thickness (represented by RVEDP) when RVEa remains too high (mean value of ~1.30 in this study) compared with its normal value range [30, 37]. Table 3 shows that the mean values of RVEDV and RVEDP in NYHA class III were increased by 65.7% and 43.1% from class II, respectively, while the increments from class I to II are 19.4% and 29.9%. The larger increase of RVESV, i.e., an 89.7% increment from class II to III compared with 31.1% from class I to II, is concomitant with this heterometric change in RV function. In addition, the failed RV pulmonary coupling in heterometric adaptation also leads to the decrease of RVEF. Though RVEDV has been substantially increased in this adaptation process in order to maintain the RVSV, the accelerating increase of RVSV (33% increase from class II to III compared with 7% from class I to II) suggested that the function of this heterometric adaptation was deteriorating. In fact, the RVSV was closely associated with the limit of significance (*p* = 0.171, data not shown) with the grouping of classes I/II and class III (Table 4). Therefore, we infer that the transition from class II to III is probable at the upper intermediate or late stage of RV heterometric adaptation.

The normal right ventricle is a thin-walled and crescent-shaped flow generator and is generally unable to afford a brisk pressure increase from the pulmonary artery [38]. PH-induced RV HF is usually defined as a consequence of elevated RV afterload (RVEa) [28]; this corresponds to our finding that RVEa correlated with the NYHA classification of classes I, II, and III, residing on the edge of significance (*p* = 0.095, data not shown). In our results, RVEDV, RVESV, RVEDP, and RVESP correlated significantly (*p* < 0.05) with NYHA classes I, II, and III (Table 4). In reality, RV enlargement always occurs much earlier in the course of PH, brought on by the increase of RV wall stress, compared with LV which can be interpreted mechanically by the fact that RV wall stress is greater for a comparable pressure increase as result of the smaller RV thickness [28, 39, 40]. This supports and explains our finding that the four aforementioned measurements positively correlated with NYHA classification (Table 4).

In the ROC analysis result (Figure 3), the RVEa and RVESP perform best among all PV loop measurements in predicting the level of PH according to NYHA classification (p_RVEa_ < 0.05; p_RVESP_ < 0.01). These two measurements correlate significantly with the grouping of class I vs classes II/III (Table 3).

### Limitations

There are several limitations to the present findings. First, this study was limited by the relatively small size of patients (*N* = 36) participating in the research. In addition, there was also a limited range of NYHA classes represented in the patient sample, i.e., with only five patients in class I and eight in class III, and null in class IV. However, the study was heavily dependent on outpatient environment and focused on less symptomatic PH patients; in such a context, this distribution of classes is common and acceptable.

## CONCLUSIONS

The performance of these PV loop objective measurements indicates they are a possible alternative to the more qualitative NYHA classification system. RVESP and RVEa show a significant association with NYHA classifications (I and II/III). This finding is of capacity to distinguish the asymptomatic class I and mildly symptomatic class II, in which the traditional and prevailing NYHA always gains immense criticisms. And RVEDV, RVESV, and RVEDP have a significant association with NYHA classification (I/II and III). This finding is providing a more serious, reliable and reproducible assessment for symptomatic PH patients who is entering or may enter the end-stage. These results suggest PV loop measurements’ promising role in assessing functional capacity in progressive but less symptomatic PH patients.
